# The value of real-world testing: a qualitative feasibility study to explore staff and organisational barriers and strategies to support implementation of a clinical pathway for the management of anxiety and depression in adult cancer patients

**DOI:** 10.1186/s40814-020-00648-4

**Published:** 2020-07-29

**Authors:** Liesbeth Geerligs, Heather L. Shepherd, Nicole M. Rankin, Lindy Masya, Joanne M. Shaw, Melanie A. Price, Haryana Dhillon, Colette Dolan, Gabrielle Prest, The ADAPT Program Group, Gavin Andrews, Kate Baychek, Philip Beale, Karen Allison, Josephine Clayton, Joseph Coll, Jessica Cuddy, Afaf Girgis, Peter Grimison, Tom Hack, Brian Kelly, Laura Kirsten, Toni Lindsay, Melanie Lovell, Tim Luckett, Michael Murphy, Jill Newby, Frances Orr, Alison Pearce, Don Piro, Tim Shaw, John Stubbs, Rosalie Viney, Fiona White, Jackie Yim, Phyllis Butow

**Affiliations:** 1 Psycho-oncology Co-operative Research Group (PoCoG), The University of Sydney, Sydney, NSW Australia; 2 Centre for Medical Psychology and Evidence-based Decision-making (CeMPED), The University of Sydney, Sydney, NSW Australia; 3 Faculty of Health Sciences, The University of Sydney, Sydney, NSW Australia; 4 The Kinghorn Cancer Centre, Sydney, NSW Australia

**Keywords:** Implementation science, Health services research, Clinical pathway, Feasibility study, Psycho-oncology, Barrier analysis, Qualitative analysis

## Abstract

**Background:**

Effective translation of evidence-based research into clinical practice requires assessment of the many factors that can impact implementation success. Research methods that draw on recognised implementation frameworks, such as the Promoting Action Research in Health Services (PARiHS) framework, and that test feasibility to gain information prior to full-scale roll-out, can support a more structured approach to implementation.

**Objective:**

This paper presents qualitative findings from a feasibility study in one cancer service of an online portal to operationalise a clinical pathway for the screening, assessment and management of anxiety and depression in adult cancer patients. The aim of this study was to explore staff perspectives on the feasibility and acceptance of a range of strategies to support implementation in order to inform the full-scale roll-out.

**Methods:**

Semi-structured interviews were conducted with fifteen hospital staff holding a range of clinical, administrative and managerial roles, and with differing levels of exposure to the pathway. Qualitative data were analysed thematically, and themes were subsequently organised within the constructs of the PARiHS framework.

**Results:**

Barriers and facilitators that affected the feasibility of the online portal and implementation strategies were organised across eight key themes: staff perceptions, culture, external influences, attitudes to psychosocial care, intervention fit, familiarity, burden and engagement. These themes mapped to the PARiHS framework’s three domains of evidence, context and facilitation.

**Conclusions:**

Implementation success may be threatened by a range of factors related to the real-world context, perceptions of the intervention (evidence) and the process by which it is introduced (facilitation). Feasibility testing of implementation strategies can provide unique insights into issues likely to influence full-scale implementation, allowing for early tailoring and more effective facilitation which may save time, money and effort in the long-term. Use of a determinant implementation framework can assist researchers to synthesise and effectively respond to barriers as they arise. While the current feasibility study related to a specific implementation, strategies such as regular engagement with local stakeholders, and discussion of barriers arising in real-time during early testing is likely to be of benefit to all researchers and clinicians seeking to maximise the likelihood of long-term implementation success.

## Background

Evidence-based interventions are associated with improved patient outcomes and greater cost-effectiveness of care [1], but despite careful planning, are not always successfully implemented in the real world [2]. The discipline of Implementation Science seeks to identify key factors that facilitate uptake of evidence-based interventions into clinical practice [3]. A range of implementation frameworks now exist to guide researchers [4], from determinant frameworks that focus on factors influencing implementation outcomes, such as the Promoting Action Research in Health Services framework (PARiHS) [5], to implementation theories, such as the Normalisation Process Theory [6], to those that focus on evaluating the implementation process, such as the RE-AIM framework [7]. A carefully selected framework can provide a strong theoretical basis from which to approach assessment of implementation, from the early stages of pilot and feasibility testing, to long-term sustainability.

To improve the likelihood of successful implementation, recent guidance from the United Kingdom Medical Research Council recommends early assessment of the feasibility of key components underlying the implementation process prior to a full evaluation [8]. This small scale real-world testing allows researchers to observe the process of translating research into practice, identify barriers that may not have been evident during the development phase, and develop strategies for facilitating smoother implementation in the long-term [9]. Within implementation science, such feasibility studies seek to assess aspects of the implementation process rather than the intervention. Whilst many studies report lessons learned by researchers during pilot and feasibility phases, the systematic review indicates that fewer have collected formal data on the views of staff experiencing the implementation, particularly in relation to clinical pathways [10]. Frontline staff are intimately involved with the implementation of any new intervention, and can therefore contribute vital information about the feasibility of key components.

This study aimed to address this gap by eliciting staff perspectives on individual and organisational barriers to the implementation of an online portal to operationalise a clinical pathway for the management of anxiety and depression in adult cancer patients (the ADAPT CP [11]). Specifically, our objectives were to identify staff perceptions of the strategies underlying the implementation of the ADAPT CP including the online portal, the training and the implementation support, to assess their feasibility within the planned cluster randomised trial and identify factors that would facilitate successful uptake of the CP in routine care. Planning and analysis were guided by the PARiHS framework, a determinant framework that was chosen due to its grounding in health services research, its generation from the clinical evidence base and its careful attention to real-world elements of context and facilitation, which may be critical to understanding the issues that arise in pilot stages [5]. The PARiHS posits that successful implementation is likely to occur when the evidence is robust, the context is supportive, and the intervention is appropriately facilitated [12].

### The study context

The ADAPT CP was developed in response to the recognised high rates of anxiety and depression in people with cancer and the lack of standardised screening, assessment and management processes [11, 13]. It incorporates iterative screening, triaging to five levels of anxiety/depression with specific recommendations regarding the content, process and intensity of care, and the ability to be tailored to individual centres’ available resources, referral networks and preferred models of care. Development was guided by evidence review, wide stakeholder consultation, and a Delphi consensus process involving > 80 experienced multi-disciplinary clinicians [13]. Guided by a barrier and enabler analysis [14], resources and strategies were incorporated into the ADAPT CP and its planned implementation. The barrier and enabler analysis was carried out with 12 multi-disciplinary health professionals from 8 medical and allied disciplines, who reviewed and provided feedback on potential barriers and enablers to implementation of the new clinical pathway; their responses informed development of resources and strategies for the full trial and the strategies tested within this feasibility study [14].

A core resource is the online portal (the ADAPT Portal), which systematically operationalises the ADAPT CP and carries out a range of automated processes for screening, alerts and referrals. Alongside the Portal, the ADAPT implementation strategies include awareness campaigns, academic detailing, reporting and technical support [15]. This single-site study was designed to assess the feasibility and acceptability of the ADAPT CP implementation strategies in routine clinical practice, including the ADAPT Portal, in order to refine these strategies for use during a large implementation-focused cluster randomised trial to implement the ADAPT CP in 12 cancer services across New South Wales, Australia [15].

## Methods

### Design

This feasibility study used a non-randomised, cross-sectional design, collecting qualitative data at a single site. Recent research has highlighted the important role of qualitative research in providing greater depth of information regarding key feasibility challenges, which may then be used to further refine the implementation strategies prior to a full trial [16]. We therefore selected this approach to meet the aims of the current study and used the consolidated criteria for reporting qualitative research (COREQ), a 32-item checklist for interviews and focus groups (see Additional file 1), to guide structure and reporting [17]. This feasibility study was designed to inform the multi-site cluster randomised trial, in which sites will be randomised to different levels of implementation support—full details of the main trial are available in the published protocol [15].

### Setting and procedure

Cancer service staff at a large, tertiary, public hospital participated in this feasibility study. This process included lead team engagement meetings to clarify roles and processes, followed by training and use of the ADAPT Portal and resources for 5 months, with system support. Afterwards, fifteen staff took part in semi-structured interviews either face-to-face at the service in a private room, or via telephone at a time convenient for participants. Interviews were audio-recorded and transcribed verbatim.

### Participants

Staff were eligible to participate if they had been involved in any way with the ADAPT Portal and implementation strategies. Staff were purposively sampled across clinical and non-clinical roles and invited to participate via email. All agreed to participate. The study was approved by the Human Research Ethics Committee of the participating healthcare institution.

### Methodological orientation and interview guide

The interviews comprised questions designed to assess all elements of the implementation strategies and overall staff experience of the implementation process, informed by the PARiHS framework and our recent systematic review of hospital-based implementation barriers and facilitators [10]. The interview guide was pilot tested by two authors (LG and PB). Sample interview questions are shown in Additional file 2. Participants were informed that the interviewer (LG) was a clinical psychologist familiar with the ADAPT CP and Portal, but not involved in the study process at the health service, and that their data would be kept confidential and reported only in summary format.

### Data analysis

NVivo10 qualitative data analysis software was used for data management and analysis. Thematic analysis was used to identify key themes regarding barriers and facilitators to implementation. A subset (20%) of transcripts were coded separately by four authors (LG, PB, NR and HS) to identify preliminary concepts, with iterative discussion to refine codes and sub-codes. Following this, LG coded the remaining transcripts. Similar concepts were grouped into themes; patterns between themes and subthemes were mapped into a thematic schema, with illustrative quotes. In line with qualitative research standards [18], reflection and reflexivity were used to mitigate any biases. Summaries of the findings were sent to a subset of participants for review; all affirmed the findings were accurate. Themes were then grouped in relation to the PARiHS framework.

## Results

### Participant sample details

Fifteen multidisciplinary staff, including psychologists, social workers, doctors, nurses, administrators, and managers participated. The sample comprised both full-time and part-time staff, who had been in their current role an average of 3 years (range 5 months to 7 years). Interviews ranged in length from 16-50 min (average 25 min).

### Qualitative analysis

We identified eight key themes that impacted the implementation process and feasibility of the implementation strategies during the study: staff perceptions of the intervention, culture, external influences, attitudes to psychosocial care, intervention fit, familiarity/exposure, engagement and burden (see Table 1). Themes were mapped to the PARiHS domains of evidence context and facilitation, allowing us to situate findings within an established implementation science framework, while at the same time illustrating the aspects that held most relevance to this context. Themes are presented under each PARiHS domain.

**Table 1:** Qualitative themes as related to the PARiHS framework

Evidence	Context	Facilitation
Staff perceptions	Culture	Intervention fit
	External factors	Familiarity/exposure
	Attitudes to psychosocial care	Burden
		Engagement

#### Evidence

Staff perceptions of the evidence underlying ADAPT CP, as presented during training and awareness campaigns, elicited both facilitators and barriers to implementation. In general, the evidence-base behind the ADAPT CP was well-recognised and accepted by staff, acting as a motivator and facilitator for implementation. However, staff acceptance of the evidence of *need* for the ADAPT CP in their service was lower, which at times acted as a disincentive for implementation.

##### Staff perceptions of the intervention

Awareness that the ADAPT CP was being implemented as part of an evidence-based research program, and comprised reputable and recognised resources acted as a key facilitator to implementation:

I like the fact that it’s linked up with the CRUFAD [Clinical Research Unit for Anxiety and Depression] cancer program [an online cognitive behavioural program]. (Participant 7)

The fact that this is being done in the protective shell of a research program is very helpful …. many may be sceptical about the benefits of some of these programs for their patients, and if it’s couched in a study way, they’re much more likely to accept that it needs to be looked at. (Participant 14)

Staff generally perceived the ADAPT CP would improve the mental health outcomes of their patients, especially noting the benefits of more formal processes for care, ensuring that screening occurred consistently and patients were matched with specialist staff with the appropriate skills and abilities:

“From the people I’ve spoken to, everyone said, oh, it’s a really good idea, … we don’t want to miss those patients who might not necessarily obviously need support.” (Participant 3)

“So, it’s nice to get a referral that’s related to their psychological wellbeing …because, we are trained in that. So that’s been good. Refreshing.” (Participant 3)

However, staff responses to the ADAPT CP and Portal were also shaped by local knowledge of their existing system, which they perceived to be highly effective, thus making the ADAPT CP somewhat redundant in their setting:

I can see how it would be helpful for maybe smaller hospitals or hospitals that don’t have good triaging in place, but here, it already feels like the needs are being met. (Participant 7)

Staff suggested that low perceived evidence of need could be addressed by providing more information about the evidence-base and rationale for screening and care, and highlighting the positives of the ADAPT CP through early sharing of data as an additional strategy:

The rationale can often make it easier for staff… it would be, this is why we’re doing it… Instead of, we’ve got to do it. (Participant 9)

If they can see that their action using the Portal has resulted in a benefit for at least some of the patients … I think that they will be very keen to continue it. (Participant 14)

#### Context

Service culture, external influences and attitudes to psychosocial care all had an impact on how the ADAPT Portal and CP strategies were received during the implementation process.

##### Culture

Strong values around quality patient care led staff to engage proactively with the ADAPT Portal despite barriers:

*“People are always wanting to provide the best that we can for the patients.” (Participant 1)*

Staff also reported a culture of supporting and drawing on each other to address any implementation barriers:

If I got a referral now I’d just speak to the team or the ones who have used it [ADAPT Portal] … I’d go to them first. (Participant 4)

A key culture-based barrier related to communication about service goals for the implementation, and a perceived lack of shared decision-making within the service. Some clinical staff felt the implications of implementation for their workload had not been adequately considered, and noted that greater engagement by managerial and leadership staff with their views, needs and resources could have overcome these issues:

I think probably rather than agreeing to it straight away, it should have involved more of the team approach… I don’t think it really took into account the implications that it would have on staffing and the increase in time. (Participant 7)

Managerial staff reported the reason for participating in the ADAPT feasibility study was to improve communication and consensus across different roles and teams, to overcome a siloed model of care and foster greater multidisciplinary action and clearer referral pathways:

The project came at a very good time, because we’d been trying to implement ...a more integrated approach … our psychosocial care staff were ...very segmentalised, with a bit of a disconnect between the three roles of nursing, social work, and clinical psychology, ... and we found that referrals to any of those three categories of psychosocial support from medical staff were inconsistent from the point of view of reason for referrals or normal sort of pathways. (Participant 13)

These motivations for taking on the study were not always communicated across the service, leading to confusion about the true purpose of service involvement, impacting user acceptance of the process and creating barriers to successful implementation.

##### External influences

The broader external context in which the service existed also had bearing on how staff responded to the implementation strategies and processes. Some staff believed that their service had participated in the study to influence public perception by appearing more research intensive, which reduced motivation to engage:

I’m sure there’s something where [the service] want to look like they’re involved in this cutting-edge research or, you know, rather than is there actually a need for it here. (Participant 7)

Another external barrier was the perception that intervention sustainability after research is completed is dependent on external funding sources, fostering a belief that interventions such as ADAPT CP were often short-term, rather than leading to sustained and adequately resourced clinical change:

If the program shows that this is very useful and that people should have this, …, well where is it going to come from? Sure, studies like this enable us to lobby government for more funding … but it’s very difficult in practice to get that kind of funding. (Participant 14)

##### Attitudes to psychosocial care

Where staff felt that managing anxiety and depression fitted with their existing role, integration of the ADAPT Portal and ADAPT CP was easier:

From day one I’ve always been aware that there are needs around these patients’ anxiety, depression, and I’ve seen it, so for me personally it hasn’t been really difficult. (Participant 2)

The response to the implementation process was also stronger when staff believed the ADAPT Program could increase staff skills and confidence to address mental health issues:

One of the key benefits that I see from a project like this, is empowerment of the nursing and allied health staff; that it’s okay yes, in fact, more than okay, it’s your job to refer someone... And so that’s …an outcome that I think is supported by this institution …to make sure that everybody feels empowered to say something if they’re concerned about a patient under their care. (Participant 14)

#### Facilitation

Themes related to facilitation suggested a need for further tailoring of ADAPT implementation strategies to address issues of intervention fit, familiarity with the pathway, sense of burden and engagement.

##### Intervention fit

A key implementation strategy designed to support the ADAPT CP was the ADAPT Portal, and analysis revealed several feasibility issues related to lack of fit and duplication with existing systems and procedures in terms of IT, communication and work patterns:

[It] kind of double dips because we have all electronic medical records, that we make our referrals through and then obviously have the ADAPT Portal which we were taking the referral through…so it was two separate systems. (Participant 10)

Duplication was quickly resolved by discussion with the research team and amendment of the ADAPT Portal to integrate into existing care workflows and electronic medical records, resolving this issue for participants:

On MOSAIQ® they would say…already linked in with psych. So the nurses would know not to re-screen. (Participant 7)

Intervention fit to patient abilities also concerned some staff, who were aware that some of their patients lacked the skill to use or access technology. However, staff noted that when carried out in clinic, the process had been relatively smooth, indicating that the onsite Portal use was feasible:

Some patients would probably not have that technology to do it at home and [with] the assistance of the nurses are able to navigate the Portal a bit better. (Participant 2)

##### Familiarity/exposure

For some staff, lack of exposure to the ADAPT Portal created challenges to implementation, with staff noting a time lag between training and their first real use of the Portal. This was partially related to effective existing referral networks making identification of such patients via the ADAPT Portal infrequent:

By the time we got a referral we thought, oh how do we do this? How do we log in? What do we do? (Participant 3)

Staff, especially those not involved in the implementation lead team, were not always clear about their roles, indicating that information had not always trickled down adequately. These staff had often had less intensive training, which compounded their sense of uncertainty.

I don’t feel really confident about it now… I wasn’t sure what else my responsibility was. (Participant 1)

However, staff believed that they would become more comfortable with ADAPT Portal processes over time:

It’s just general awareness, just a matter of like, really getting in the habit of doing it. (Participant 2)

To address these concerns, repeat training and on-the-spot training were recommended in addition to local prompts, summary sheets and simple reminders with step-by-step processes as helpful tools to improve exposure and confidence in their roles. To assist with familiarity and exposure, staff also proposed that more than one trainer and champion could be present at the services in the early stages to reinforce the rationale and process of the ADAPT CP and Portal:

I think it would be nice for people to come back and check periodically to see … what’s going on, you know? Is there anything we need to do… and just explain to us why patients are doing this. (Participant 9)

##### Burden

Facilitation was also impacted by a sense that implementation of ADAPT would result in an increased workload for staff, with staff shortages and heavy existing workloads contributing to a sense of burden. Restricted time had flow-on effects, impacting staff ability to attend training to follow proposed processes:

It’s not that it takes that long, but ...it’s like obviously an extra thing that you’re being asked to do. (Participant 1)

Staff time was probably the biggest [barrier]… finding time, for the training of everyone. (Participant 6)

These issues were resolved during facilitation meetings between the research team and staff, in which the pathway was tailored and modified to better fit with the flow of the service:

So that did take a little bit of pushback for them…but ultimately, … we got there in the end. (Participant 11)

This sense of additional burden was also related to an overload of new information, both in relation to ADAPT and other ongoing initiatives. However, this was mostly perceived as a usual and expected part of change-management:

I think there was underlying sense of …oh god, you know, there’s another system we’ve got to use. (Participant 13)

So I did notice a little bit of tension but, I think, that’s natural whenever you’re introducing something new. (Participant 6)

Finally, it was noted that despite this apprehension, initial concerns about increased workload were not realised once the study commenced:

When it, rolled out, we were all panicking and we thought…how are we going to take on all these new patients? We were really surprised that we only got a couple. (Participant 3)

##### Engagement

As noted in the “Culture” section, staff felt they had been inadequately engaged in the early stages, and as such lacked a sense of ownership or connection to the process, despite the ADAPT implementation strategies designed to target these issues. To address this, staff highlighted the need to approach all stakeholders early to seek feedback, and to secure buy-in from key service members, such as doctors:

Ideally it would be meeting with the researchers and hearing about the project, being told that it is a choice … being told why [the service] want to do it, … allowing clinicians to feel as though they played a part in the decision. (Participant 7)

I think it needs to be presented to the Department Heads…needs to get buy in from the clinicians, the doctors. (Participant 10)

Additional support as an implementation strategy from both the external facilitators and internal team members were also proposed:

There should be someone from the group who wanted to start this program to continue to be the other presence, and maybe just to maybe get a session from the educator, just to remind everyone why we’re doing this. (Participant 2)

However, others had mixed attitudes toward the role of researchers as facilitators. While all staff reported liking and respecting the researchers, some noted a lack of shared terminology, and poor perceived researcher understanding of the reality and priorities of clinical care:

Yeah, it took a little while to get to that point, because the research team… their expectations of what we were capable of doing and what we should be doing was a little bit unrealistic. (Participant 11)

Finally, staff indicated that to support engagement at the service level, dissemination of information and training needed to be better tailored to meet the needs of part-time and shift-workers. Timetabling and scheduling of education to fit staff shifts were proposed as an implementation strategy to address this problem:

It happens all the time and we need to get them remember that we’re part-time and sometimes you don’t know that the new way of doing something, until you are getting pulled up for not doing it. (Participant 1)

## Discussion

The current study sought to explore the feasibility and user acceptance of a range of implementation strategies to support the ADAPT CP, including the ADAPT Portal. While the benefits of evidence-based clinical pathways for patients are well-recognised, our real-world feasibility testing of the ADAPT Portal and ADAPT CP implementation strategies demonstrates the importance of evidence, contextual, and facilitation factors when implementing a stepped-care intervention in a clinical service. Early identification and assessment of barriers in these areas provide vital information, allowing researchers to tailor strategies in order to resolve real-world challenges prior to full-scale rollout. Our qualitative analysis identified eight distinct themes where barriers arose in relation to the Portal and implementation process. Mapping these themes to the three domains of the PARiHS framework allowed us to situate them within a recognised implementation science framework and further synthesise this information for practical use.

The first area that impacted user experience of the implementation process was evidence. The PARiHS framework posits that implementation is most likely to be successful when evidence for the intervention is robust: not only traditional notions of evidence arising from randomised controlled trials (codified evidence), but also other types of evidence that inform clinical practice, including practitioner expertise, patient experiences, and local information (non-codified evidence) [19]. In the current study, both codified and non-codified evidence strongly influenced the way staff perceived implementation of the ADAPT Portal and CP. Staff were motivated by known codified evidence supporting the efficacy of screening and management of anxiety and depression. However, local perceived evidence regarding the efficiency of current processes for managing anxiety and depression led them to view the ADAPT Portal and CP as redundant. These findings highlight the need for implementation scientists to explore local forms of evidence and how these may shape perceptions and create avoidable barriers to implementation. It is possible that locally collected pre-implementation data (such as audit and review) on the success of current service approaches prior to the introduction of the ADAPT CP could have been useful in shifting views. Indeed, studies of clinical pathways in other areas suggest that provision of quantifiable outcomes in pre-implementation can be a key strategy to generating effective dialogue with clinical staff [20]. Audit and feedback data from the ADAPT Portal could also help to provide an additional source of real-time, concrete evidence of changes resulting from the implementation over time. The main cluster randomised trial of ADAPT will integrate audit and feedback as a strategy to support continual awareness of the impact of the implementation.

Contextual factors of service culture, external influences and attitudes to psychosocial care also influenced acceptance of the implementation process and strategies. Staff acknowledged diversity in the goals of different teams within their service, at times creating dissonance and barriers to implementation. This is consistent with the PARiHS framework view of health-care services as multiple interconnected systems, whose interactions can create friction and complexity [21]. To overcome these barriers, staff suggested that management and research staff be more transparent about implementation goals and intended outcomes. While the PARiHS framework highlights the role of leadership in driving change [21], researchers may need to engage not only with leaders, but staff at all levels, particularly those implementing strategies on the ground. This would ensure that diverse perspectives are heard and reflected in intervention design and selection of implementation strategies. Researchers may in turn be able to support leaders and champions to create a unified approach and commitment to implementation, possibly even creating cultural change beyond the intervention itself. The cluster randomised trial will adopt a more extended engagement process, allowing researchers to understand the unique dynamics of each service and connect with staff in all roles. Staff also perceived that their service was impacted by a range of external influences, such as public perceptions and funding requirements, which they felt could impact on sustainability of the implementation. This is consistent with findings showing that external demands that create a sense of threat or uncertainty have a significant impact on innovation implementation in teams, reducing openness to change [22]. Given the positive culture reported around patient care, explaining more clearly how the clinical pathway tied into this value could have improved motivation and acceptance. Additionally, staff who felt that involvement with the ADAPT CP could provide an opportunity for strengthening their skills and documented experience in psychosocial care were more receptive to the implementation process. Emphasising these potential unexpected staff-related benefits of an intervention could be part of an implementation strategy where the culture is expected to benefit from this.

Finally, the importance of facilitation was evident within the themes of intervention fit, familiarity, burden and engagement. In the current study, tailoring the implementation strategies and systems supporting the ADAPT Portal, so that it complemented rather than replaced existing processes, were key to addressing issues. Adjustments to the strategies of training and education, such as repeat sessions to meet the needs of part-time workers and provision of in-person service support, were also integrated for the cluster randomised trial. The PARiHS framework highlights the importance of engaging end users as part of the facilitation process. While engagement prior to implementation is often discussed in the public health implementation literature [23, 24], it has only recently begun to be explored in relation to hospital service implementation [25]. Early connection with service staff has a powerful role in establishing relationships, providing insight into context, and highlighting the key facilitation needs of the service. Our findings reinforce the importance of a well-considered engagement strategy to ensure a strong sense of staff ownership of the intervention and the implementation process. To assist this process during the cluster randomised trial, an extended engagement process has been adopted, allowing the research team more time to understand the service context and support the service champions to implement the ADAPT CP.

This real-world feasibility study was helpful in fine tuning the ADAPT Portal and implementation strategies for the subsequent cluster randomised trial, which commenced in 2017, with final data collection envisaged for 2020 [15]. Conversations about terminology, hospital processes and flow of systems allowed the facilitators and staff to develop a shared language and pre-empted some barriers during the study. The established relationship also allowed for open communication between staff and the research team during implementation, meaning information about challenges was quickly relayed, and could often be collaboratively resolved. Finally, the role of the research team as an external facilitator is garnering increasing attention in implementation science [26]. Qualitative research has long acknowledged the role of reflexivity [18] and it is highly relevant in implementation research, where researchers frequently spend extended amounts of time in the setting, supporting implementation processes and responding to barriers. They may be a crucial component in the change process [26]. Researchers’ awareness of how they may influence implementation outcomes and their impact on sustainability is an area ripe for further investigation.

### Strengths and limitations

The strengths of the study include the collection of formal qualitative data from service staff, which provided depth of information about the implementation experience, the high level of methodological rigour applied to data collection, synthesis and analysis and the use of a widely used implementation framework specifically developed for health services. A number of study limitations must also be considered. The small sample of participants meant that only 1-2 participants from each role type were interviewed. The generalisability of our findings is impacted by the specialist setting of oncology. However, many of the issues raised were not limited to oncology but to clinical pathway implementation in general, suggesting our findings may have relevance to other clinical services. This feasibility study was conducted in an urban service, potentially impacting generalisability to regional or rural settings.

### Future directions and clinical implications

Our findings highlight the need for early engagement with stakeholders, and the use of co-designed implementation strategies to effectively transfer evidence-based approaches into real-world settings. Feasibility testing of resources, programs and strategies provides early insight into changes that may lead to greater implementation success long-term [27]. To fully understand and effectively assess context and evidence, future implementation studies should incorporate use of quantitative data, to allow triangulation with qualitative results, adding rigour and breadth to early stage findings. Recent work with the PARiHS framework poses a two-part process, in which context and evidence are assessed first, and then tailored facilitation follows [28]. This approach is likely to garner the most benefits from the framework, and the planned ADAPT cluster randomised trial will follow this approach.

## Conclusion

This study demonstrates the importance of identifying and addressing practical barriers that may emerge only during real-world testing, underscoring the importance of the small-scale testing to assess feasibility and acceptance of implementation strategies and processes. In line with the PARiHS framework, issues emerged in the current feasibility study across the three domains of evidence, context and facilitation. Our results show that clinical service staff are deeply connected to evidence shaped not only by academic research, but also by years of local, co-created knowledge of their own practice and the needs of their patients [29]. Engaging with this local knowledge, and respectfully taking time to understand the dominant cultural narratives of the service are essential to creating an implementation process that can integrate with existing practices, respond to potential barriers or insufficient resources, and connect to the values and needs of staff who will carry out key roles. Finally, the ability of the implementation team to provide a smooth process of facilitation, that addresses these issues as openly as possible, cannot be underestimated. Taking time to create a collaborative relationship between implementation researchers and clinical staff provides a firm base from which to approach the challenges of real-world implementation, in a way that increases the likelihood of longer-term success.

## Supplementary information

**Additional file 1.** COREQ Checklist. Consolidated criteria for reporting qualitative checklist (text data in table form).

**Additional file 2.** Sample interview questions from moderator guide. Text data excerpt from interview guide (table form).

## Data Availability

The datasets generated and analysed during the current study are not publicly available, as this was a qualitative study with specific goals and questions. The data have been fully analysed for this manuscript and therefore will not be available to other researchers, although the researchers are happy to consider reasonable requests via the corresponding author.

## Abbreviations

ADAPT CP: Anxiety and Depression Clinical Pathway
PARiHS Framework: Promoting Action Research in Health Services Framework
