# Supplementary material for: The value of real-world testing: a qualitative feasibility study to explore staff and organisational barriers and strategies to support implementation of a clinical pathway for the management of anxiety and depression in adult cancer patients
Source: Pilot Feasibility Stud. 2020 Jul 29;6:109. doi: 10.1186/s40814-020-00648-4 (PMC7388211; doi:10.1186/s40814-020-00648-4)
Supplement: Supplementary file 2 — Additional file 2. Sample interview questions from moderator guide. Text data excerpt from interview guide (table form). [file 40814_2020_648_MOESM2_ESM.docx]

**Additional File 2. Sample interview questions from moderator guide**

| PARiHS domain | Example question |
| --- | --- |
| Evidence | *Did the services and support information available on the ADAPT Portal help you in your role? If not, why not?* |
| Context | *How does the culture of your workplace respond to new technologies in general? And the ADAPT Portal specifically?* |
| Facilitation | *Could you have been better prepared to use the ADAPT Portal? How?* |
